# Large genetic screens for gynogenesis and androgenesis haploid inducers in *Arabidopsis thaliana* failed to identify mutants

**DOI:** 10.3389/fpls.2015.00147

**Published:** 2015-03-12

**Authors:** Virginie Portemer, Charlotte Renne, Alexia Guillebaux, Raphael Mercier

**Affiliations:** ^1^INRA, UMR1318, Institut Jean-Pierre BourginVersailles, France; ^2^AgroParisTech, Institut Jean-Pierre BourginVersailles, France

**Keywords:** gynogenesis, androgenesis, *Arabidopsis thaliana*, haploid, EMS mutagenesis, genetic screen

## Abstract

Gynogenesis is a process in which the embryo genome originates exclusively from female origin, following embryogenesis stimulation by a male gamete. In contrast, androgenesis is the development of embryos that contain only the male nuclear genetic background. Both phenomena are of great interest in plant breeding as haploidization is an efficient tool to reduce the length of breeding schemes to create varieties. Although few inducer lines have been described, the genetic control of these phenomena is poorly understood. We developed genetic screens to identify mutations that would induce gynogenesis or androgenesis in* Arabidopsis thaliana*. The ability of mutant pollen to induce either gynogenesis or androgenesis was tested by crossing mutagenized plants as males. Seedlings from these crosses were screened with recessive phenotypic markers, one genetically controlled by the female genome and another by the male genome. Positive and negative controls confirmed the unambiguous detection of both gynogenesis and androgenesis events. This strategy was applied to 1,666 EMS-mutagenised lines and 47 distant *Arabidopsis* strains. While an internal control suggested that the mutagenesis reached saturation, no gynogenesis or androgenesis inducer was found. However, spontaneous gynogenesis was observed at a frequency of 1/10,800. Altogether, these results suggest that no simple EMS-induced mutation in the male genome is able to induce gynogenesis or androgenesis in *Arabidopsis*.

## Introduction

In sexual reproduction, the fusion of the male and female haploid gametes leads to the formation of a diploid embryo. Both parents contribute equally to the nuclear genome of the embryo. In contrast, the cytoplasmic genome (mitochondrial and chloroplastic), is solely from female origin (Berger et al., 2008). *In situ* gynogenesis and androgenesis are two deviations of sexual reproduction. Gynogenesis leads to embryos that exclusively originate from the female genetic background, with no contribution of the male in the embryo’s genome, even if a male gamete is required to stimulate embryogenesis. This differs from another mode of reproduction, parthenogenesis, where embryogenesis occurs spontaneously, in absence of a male gamete. Conversely, *in situ* androgenesis leads to the development of embryos that contain only the nuclear male genetic background, with no contribution of the female to the nuclear genome of the embryo. In both *in situ* gynogenesis and androgenesis, the cytoplasmic genome is of female origin.

Obligate gynogenesis is the natural mode of reproduction for some vertebrate species, such as some salamanders or fishes. In these unisexual organisms, males do not exist. Females produce diploid eggs and the male stimulation is performed by the sperm of related species (Neaves and Baumann, 2011). *In situ* androgenesis seems to be rare in nature (Seguí-Simarro, 2010), used for example by some clam families, the Mexican axolotl Siredon, and the Cyprus *C. serpervirens* for which another Cyprus species acts as a surrogate mother (Pichot et al., 2008).

Gynogenesis and androgenesis are of great interest for plant breeders because genome-wide homozygosity can be achieved in a single generation, reducing the time requirements of breeding programs (Dunwell, 2010; Germanà, 2011). The elimination of one genome parent leads to haploids which can then be diploidized by a step of chromosome doubling. Moreover, androgenesis can be useful to improve the cytoplasmic male sterility (CMS) system (Budar et al., 2001). The main issue of this system is the introgression of a selected nuclear genome into a male sterile line that is under cytoplasmic control. A current method is carried out by several backcrosses that are time consuming. Efficiently using androgenesis, as the cytoplasmic genome remains from female origin, only one cross is necessary to transmit CMS. In addition *in situ* androgenesis or gynogenesis have been used to create a method of clonal reproduction through seed (Marimuthu et al., 2011).

For decades various methods to induce artificial gynogenesis and androgenesis in many crop species have been exploited. Biotechnological *in vitro* approaches such as anther cultures and isolated microspore cultures are widely used to produce doubled haploids in many species. It should be noted that in the case of *in vitro* androgenesis, the mitochondrial, and plastidial genome has a male origin, in contrast to *in situ* androgenesis. For gynogenesis, ovule, ovary, and flower culture, with or without the use of mentor or irradiated pollen, is used to produce gynogenic doubled haploids in some species (Bohanec, 2009; Seguí-Simarro, 2010; Germanà, 2011). Another method is to induce *in situ* gynogenesis/androgenesis through interspecific crosses. The most documented examples are *Triticum aestivum* ×*Zea mays* (Laurie and Bennett, 1988) and *Hordeum vulgare* ×*Hordeum bulbosum* crosses (Kasha and Kao, 1970). Although androgenesis has been reported in a few cases in barley (Kasha and Kao, 1970; Lange, 1971; Finch, 1983), gynogenesis is more common (Houben et al., 2010). Irradiated pollen can also be used to induce *in situ* gynogenesis (Chat et al., 2003; Froelicher et al., 2007). Finally, specific lines that induce *in situ* gynogenesis or androgenesis following intraspecific crosses have been also reported in the literature, notably in maize (Kermicle, 1969; Eder and Chalyk, 2002; Zhang et al., 2008). This trait appears to be under genetic control. For example, the *gynogenesis inducer 1* (*ggi1*) locus has been shown to control gynogenesis induction, and is widely used in maize breeding, but the underlying gene(s) has not been identified yet (Barret et al., 2008). Another haploid inducer in maize is the *indeterminate gametophyte* (*ig*) mutant (Huang and Sheridan, 1996; Evans, 2007). *ig* can induce both androgenesis and gynogenesis (Kermicle, 1969). This process has been used to produce plants with a male nuclear genome and a female cytoplasm genome (Kindiger and Hamann, 1993). In barley, a haploid initiator mutant (*hap*) prevents fertilization of the egg cell but not the central cell. For this reason, endosperm can be formed normally and haploid embryos containing only the female genome can be developed (Mogensen, 1982). In *Arabidopsis*, the centromeric histone *CENH3* was manipulated leading to the TailSwap (TS) and Genome Elimination (GEM) line able to stimulate both gynogenesis and androgenesis (Ravi and Chan, 2010; Marimuthu et al., 2011; Ravi et al., 2014). These lines carry a null mutation in the native *CENH3* which is rescued with one or two transgenes, respectively. The *CENH3* variant(s) are required for viability because the null mutant is lethal. The transfer of this method to crops might be difficult due to this genetic complexity. The GEM line has been used for the creation of synthetic apomixis, developed by Marimuthu et al. (2011). This method combined GEM and *MiMe* in which meiosis is turned into mitosis, to induce the production of clonal seeds. However, only ∼30% of the seeds are clones because the penetrance of the GEM line is incomplete.

Hence the improvement of *in situ* haploid induction method, both in terms of frequency and availability in more species, would be of great interest. Here, with the aim of obtaining better knowledge of the genetic control of *in situ* androgenesis and gynogenesis, we ran a large scale genetic screen for mutations inducing these events in the model plant *Arabidopsis thaliana*.

## Materials and Methods

### Plant Material and Growth Conditions

The *A. thaliana* accession used for mutagenesis was Columbia (Col-0). The other 47 accessions (non-mutagenized) were Ms-0, Rubezhnoe-1, Kz-9, Kly-1, N7, N14, Leb-3, Altai-2, Sij-1, Shahdara, Bik-1, Ita-0, Cvi-0, Sei-0, Sah-0, Sakata, Ty-0, Ost-0, Lov-1, Yo-0, Pyl, Bur-0, Rld-2, Jea, Bla-1, Ran, Lod-2, Bozen-1a, Toufl-1, Cha-0, Are-0, Esc-0, Etna-1, Had-1b, Chab-1, Dja-1, Sorbo, Kondara, Kar-1, Bas-1, Nov-01, Rak-1, Chi-0, Bij, Keu-1, Shigu-2, and Stepn-1. Mutants for *APT* were used as female plants. These mutants are deficient for the enzyme APT [adenine phosphoribosyl transferase (EC2.4.2.7)] which confers 2FA (2-Fluoroadenine) resistance (Gaillard et al., 1998). The *apt* mutant is also male sterile, facilitating the crosses. Plants with a T-DNA insertion in *GLABRA1* (*GL1*) were used as male plants. *gl1* mutants do not have trichomes. Plants were cultivated in greenhouses with a 16 h/day and 8 h/night photoperiod, at 20^∘^C and 70% humidity.

### EMS Mutagenesis

Seeds were incubated for 17 h at room temperature in 5 mL of 0.3% (v/v) EMS. Neutralization was performed by adding 5 mL of sodium thiosulfate 1 M for 5 min. Three milliliter of water was added to make the seeds sink. The supernatant was removed and the seeds were washed three times for 20 min with 15 mL of water. The seeds were immediately sown in soil. EMS induce mutations by nucleotide substitutions which causes primarily G:C to A:T transitions.

### Oligonucleotides for PCR Genotyping and Sequencing

*APT* was amplified using primers with RT1 (5^′^-tcccagaatc-cgctaagattgcc-3^′^) and RT21 (5^′^-CTCAATTACGCAAGCAC-3^′^). Polymorphism between wild type and mutant alleles was revealed with Mva1 (Fermentas, Stockholm, Sweden) digestion at 37^∘^C for 1h. GL1 gene was amplified using primers with GL135SF (5^′^-TTCAAAGACAAATTCAAAACA-3^′^), and GL135SR (5^′^-GATTTGGCCGGTTAAGTTGAT-3^′^), and mutant allele using GL135SR, and PKYPM1 (5^′^-CGCAATGTGTTATTAAGTTGTCTAAGCG-3^′^). The DNA sequence of the coding region of the APT gene was amplified by PCR as 3 fragments from 500 to 1,200 bp which overlap. Differences between mutants and wild-type sequences were viewed using the Multalin program (http://multalin.toulouse.inra.fr/multalin/).

### Ploidy Determination

Chromosome spreads were prepared and stained with DAPI as described in Ross et al. (1996). Observations were made with a Leica DM RXA2 epifluorescence microscope using an oil PL APO 100X/1.40 objective (Leica). Chromosomes were counted on cells at mitotic metaphase.

### Experimental Design to Detect *in situ* Gynogenesis and Androgenesis Inducers

**FIGURE 1:**
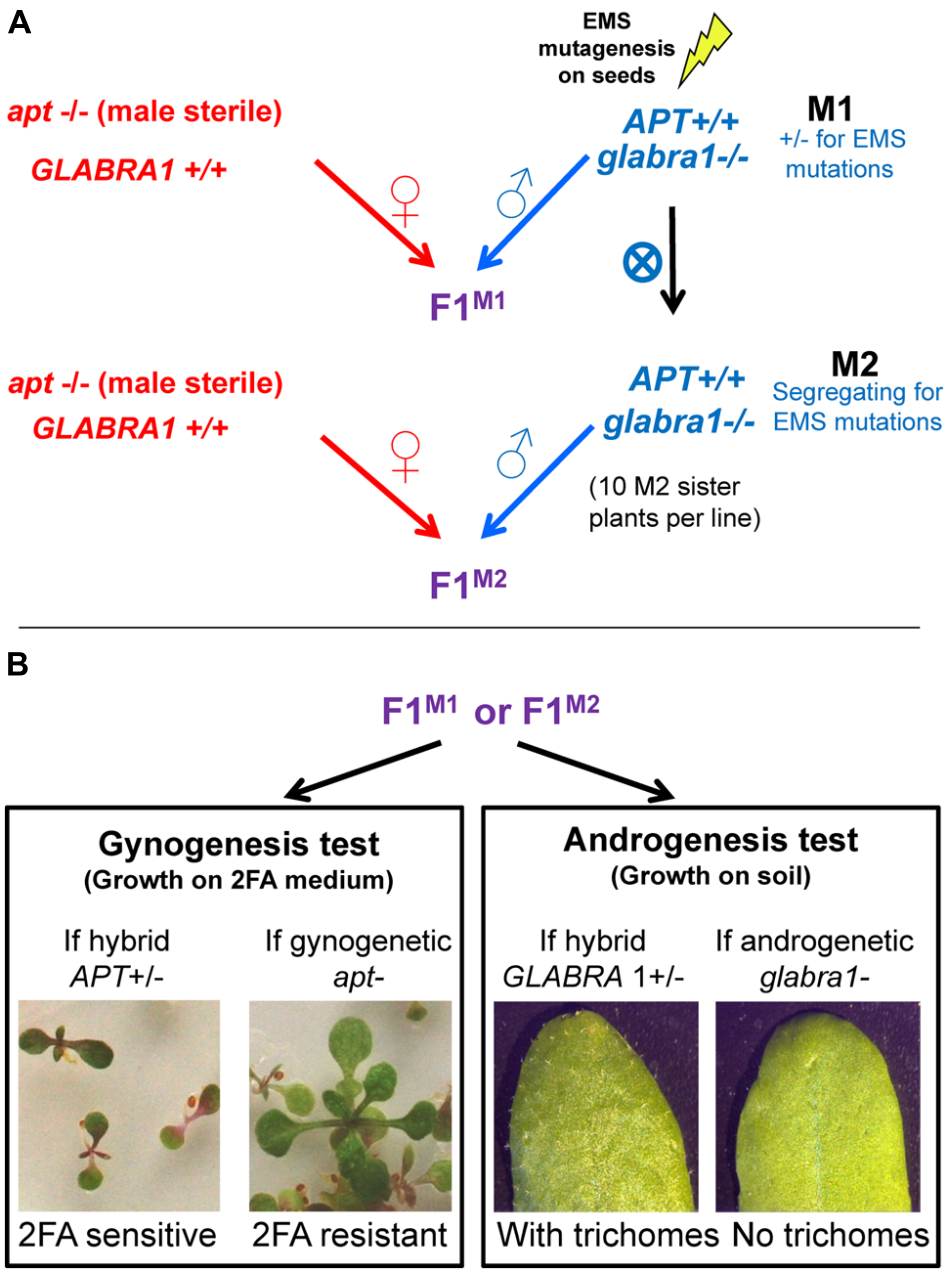
Design of the haploid inducer screen. Seeds of Col-0 strains containing the *glabra1* mutation are mutagenized to obtain M1 plants which are self-fertilized to produce M2 plants. These plants, either in M1 or M2, are crossed with an *apt* mutant **(A)**. The two mutations, *apt* and *glabra1* are recessive and confer resistance to 2FA and absence of trichomes, respectively **(B)**. In F1, if gynogenesis or androgenesis haploids appear they would lack *APT* or *GLABRA* dominant allele, respectively, and be detected because of the corresponding phenotype.

In this study, we developed a screen to detect gynogenesis and androgenesis events (**Figure 1**). The obtained plants would be haploid and would contain nuclear genetic information exclusively from either the female or the male genome, respectively. To identify such events we used two recessive phenotypic markers: 2FA resistance and absence of trichomes. The gynogenesis screen exploits the *apt* mutant which is 2FA resistant whereas the androgenesis screen is based on *gl1* mutant in which trichomes are absent on leaves. The ability of mutant pollen to induce either gynogenesis or androgenesis was tested by crossing female plants homozygous for the *apt* mutation and male plants homozygous for the *gl1* mutation.

EMS mutagenesis was applied on *gl1* seeds to produce the M1 generation (seven independent mutagenesis, **Table 1**). The screen was performed both at the M1 and M2 generations. At the M1 generation (one plant being one line), only dominant or gametophytic mutations can be detected. At the M2 generation (crossing ∼10 M2 segregated plants per line) recessive mutations can also be detected.

**Table 1:** Number of tested plants and number of *apt* mutants detected.

Mutagenesis number	Number of mutagenized plant	Number of tested plant		Number of *apt* mutant found
		in M1	in M2	
1	70	70	69	1
2	94	94	83	1
3	66	66	0	1
4	172	172	0	1
5	339	339	0	1
6	79	79	0	2
7	846	44	846	6
**Total**	**1666**	**864**	**998**	**13**

To screen for gynogenesis events, about 100 seeds (F1^M1^ or F1^M2^) were grown on 2FA medium. F1 seeds produced through regular fertilization would carry a functional *APT* allele (from the male) and thus would die on 2FA medium. In contrast, gynogenetic plants (haploid) would survive on 2FA medium because they would lack the *APT* wild type allele (**Figure 1B**, left). In parallel, to screen for androgenetic events, about 100 F1 seeds were grow on non-selective media and observed for the presence/absence of trichomes. F1s would carry a functional *GL1* allele (from the female) and would have trichomes. Androgenetic haploid plants would show an absence of trichomes because they would lack the *GL1* wild type allele (**Figure 1B**, right).

Novel *apt* mutations generated by the EMS treatment would be expected to be identified which also serves as an internal control. In that case, F1s would carry two deficient alleles for *APT* coming from both parents and 2FA resistant plants can be found in the F1s. These *de novo apt* mutations can be distinguished from gynogenetic events because (i) F1 plants are diploid, (ii) 2FA resistant plants would also been found in the self-fertilization progeny of the male parent, (iii) an additional mutation would be found in the *APT* gene. The number of *apt* mutations found in the screen can be used as a marker of the saturation level reached in the screen.

## Results

### Positive and Negative Controls

We first tested if the detection system described above is efficient to detect androgenesis and gynogenesis events, by using negative and positive controls. The negative controls were performed by crossing a Col-0 wild type plant with the two recessive markers chosen (crosses between* apt*-/- × wt [Col-0] or wt [Col-0] ×*gl1*-/-). In that case, the F1 of these crosses should be hybrids. The phenotype conferred by recessive markers should not be seen because both would be heterozygous. Indeed, neither 2FA resistant (*n* = 108) nor plant lacking trichomes were detected (*n* = 100), in the F1 of these crosses. As a positive control, we used the GEM haploid inductor line (Marimuthu et al., 2011). We produced F1 seeds by crossing* apt*-/- × GEM and GEM ×*gl1*-/-. If a gyno/androgenesis event takes place, it should be detected *via* the expression of recessive markers, as the dominant *GL1* and *APT* allele originating from the gem line would be eliminated. In these two positive controls, the two phenotypes were found: 16% of 2FA resistant plants (*n* = 158) and 58% of plants lacking trichomes (*n* = 53). These controls indicating that this screen allows the unambiguous detection of gynogenesis or androgenesis events.

### Spontaneous Gynogenetic Haploid Plants

Interestingly, 97 2FA resistant and haploid plants (determined by chromosome counting), thus from female origin, have been indeed recovered in the screen. These cases are interpreted as gynogenesis events. However, in all cases, only one such plant appeared per cross (around 100 seeds tested for any given cross in the screen). When a haploid plant was found in a cross, we further tested the corresponding line but never showed a heritable capacity to induce gynogenesis above background levels. Thus, this corresponds likely to the spontaneous apparition of haploids of female origin in *Arabidopsis*. As these 97 events were detected among ∼1,047,700 seeds (10,477 crosses ×∼100 seeds analyzed per cross), we estimate a spontaneous gynogenesis to occur at a frequency of ∼1/10,800. In contrast, no androgenetic haploid were found among ∼846,000 seeds tested.

### Number of Lines Tested and Mutation Saturation of the Genome

For the gynogenesis screen, 864 M1 lines were tested (**Table 1**). Each M1 was used to pollinate *apt* mutants, and the resulting F1s were grown on 2FA medium (∼100 plants per cross). 2FA resistant plants were found in a total of 8 independent crosses among 864. The frequency of 2FA resistant plants in each F1 ranged from 3 to 36%. For each of these 8 cases, all F1 2FA resistant plants were diploid; 2FA resistant plants were found in the selfing progeny of the male plant at a proportion ranging from 0.5 to 21%, (likely reflecting the chimeric nature of M1 EMS mutants; Koornneef, 2002); and *de novo* point mutation were found in *APT* in each of the lines (**Table 2**). Thus, we conclude that these were not gynogenesis events but EMS-induced *apt* mutations. Having found 8 *apt* mutants for 864 M1 tested suggests that the screen reached a reasonable level of saturation, and that dominant/gametophytic mutations that would confer gynogenesis are unlikely to exist. We thus stopped the M1 screen and started the M2 screen. In the M2 gynogenesis screen, 998 families were tested. Ten M2 sister plants per family were used to pollinate *apt* mutants and each resulting F1 was individually grown on 2FA medium. These 998 M2 families include 152 families used in the M1 screen and 846 families that were not screened at the M1 generation. For 8 of 998 families, ∼50% diploid 2FA resistant plants were found in at least one of the 10 F1s tested. Like in the M1 screen, these events are explained by EMS-induced *apt* mutations: 2FA resistant plants were also found in the selfing of the corresponding M2 plant (at a proportion of ∼25%), and the presence of a novel *apt* mutation was confirmed by sequencing in all cases (**Table 2**).

**Table 2:** New *apt* mutants found in the screen.

Mutant name	Fertile or sterile	Position and nucleotide change	Amino acid changes of the AT1G27450.2
gl187.9	Fertile	CHr1:9532494 C > T	P25 > S
gl23	Sterile	CHr1:9532503 G > A	G28 > R
s3pl5	Sterile	CHr1:9532608 G > A	D33 > N
gl129.11	Sterile	CHr1:9532887 G > A	G70 > D
gl865.6	Sterile	CHr1:9532887 G > A	G70 > D
s6pl30	Sterile	CHr1:9532899 G > A	G74 > D
s11pl13	Sterile	CHr1:9533395 G > A	E106 > K
gl172.10	Sterile	CHr1:9533483 G > A	G135 > D
s11pl33	Fertile	CHr1:9533485 G > A	G136 > R
s2pl6	Fertile	CHr1:9533486 G > A	G136 > E
gl392.8	Sterile	CHr1:9533599 G > A	splicing site of the 4th intron
s1pl41	Sterile	CHr1:9533621 G > A	C155 > Y
s10pl47	Sterile	CHr1:9533751 G > A	splicing site of the 5th intron

For the androgenesis screen 44 M1 plants were tested, by pollinating them with *gl1* pollen, and observing the presence/absence of trichomes on leaves of ∼100 plants of the resulting F1s. No potential androgenetic events were found among these 44 populations. We then tested 846 families at the M2 generation: 10 M2 plants per family were pollinated by *gl1* pollen and the resulting F1s were examined for the presence of trichomes (∼100 plants per F1). However, no potential androgenetic events were detected in this screen. As the same families were used for the gynogenesis screen, we know that one among the 44 M1 plant tested and six among the 846 M2 families tested contained an *apt* mutation induced by the EMS treatment, suggesting that a certain level of saturation was reached.

Finally, we tested 47 different accessions genetically distant from Col-0 to explore the possibility that natural variation could be able to induce gynogenesis. For these crosses, each accession (Ms-0, Rubezhnoe-1, Kz-9, Kly-1, N7, N14, Leb-3, Altai-2, Sij-1, Shahdara, Bik-1, Ita-0, Cvi-0, Sei-0, Sah-0, Sakata, Ty-0, Ost-0, Lov-1, Yo-0, Pyl, Bur-0, Rld-2, Jea, Bla-1, Ran, Lod-2, Bozen-1a, Toufl-1, Cha-0, Are-0, Esc-0, Etna-1, Had-1b, Chab-1, Dja-1, Sorbo, Kondara, Kar-1, Bas-1, Nov-01, Rak-1, Chi-0, Bij, Keu-1, Shigu-2, and Stepn-1) was crossed as male with an *apt* mutant as female. For each cross, about 500 F1 seeds were grown on 2FA-containing medium, but no resistant plants were found.

## Discussion

In this study, we showed that spontaneous gynogenesis occurs at a frequency of ∼1/10,800 in *Arabidopsis* crosses. In contrast, no androgenetic haploids were found among ∼846,000 seeds tested, suggesting that spontaneous androgenesis occurs, if at all, at a much lower frequency than gynogenesis. It should be noted, that these figures were obtained after manual crosses, and that we cannot exclude that they could differ in a population obtained by selfing. It is not unusual to find spontaneous haploid in different plant species like in *Brassica napus* (Olsson and Hagberg, 1955) or in maize (Chase, 1963). Spontaneous haploids of female origin were detected in maize at a proportion of 1/1,000 whereas, haploid androgenesis were found on average at 1/80,000 (Chase, 1963). In maize, spontaneous haploid seems to appear more often than in *A. thaliana*.

The screens performed here were designed to identify male genetic factors influencing the occurrence of *in situ* gynogenesis or androgenesis. However, these screens failed to identify mutations that increase the occurrence of these events to detectable levels. The controls using a known inducer line confirmed that the experimental design was able to unambiguously detect gynogenetic and androgenetic events. However, it should be noted that, as ∼100 seeds were tested per line, the screens could have missed mutations that would induce gyno/androgenetic events at frequencies lower that 5%. The mutagenized populations used in this study contained 13 independent, phenotypically detectable, mutations in *APT*. Thus *apt* mutations induced by the EMS treatment can be used as an internal measure of the mutagenesis saturation. It should be noted that not all gene are equally sensitive to mutagenesis because of their length, number of introns, or number of codons that are essential for the function of the protein. Nevertheless, it is reasonable to assume that these populations contained at least one efficient mutation in most of the *Arabidopsis* genes. However, no mutation able to induce gynogenesis and androgenesis were recovered. This may suggest that such a mutation does not exist in *A. thaliana*. However such mutations may be very specific (e.g., gain of function, separation of function, or specific levels of residual activity) and then much less frequent than simple knock out/knock down. For example, it is possible that a single mutation in the centromeric histone CENH3 gene could recapitulate the subtle equilibrium observed in the TS/GEM lines. Indeed, a null mutation in CENH3 histone is lethal, while the TS/GEM lines contain modified versions of *CENH3* (with GFP fusion and/or replacement of the CENH3 tail by the H3 tail). It appears that chromosomes loaded with these versions of CENH3 are able to segregate properly at mitosis, ensuring plant viability, but that they are eliminated when put in competition with chromosomes loaded with wild type CENH3, leading to genome elimination (Ravi and Chan, 2010; Marimuthu et al., 2011; Ravi et al., 2014). We can speculate that a single mutation in CENH3 could recapitulate the required level of CENH3 functionality. However, the screen we used here, that cannot be increased in size indefinitely as it relies on manual crosses, may very well have missed such subtle mutations.

In addition, our screen was designed to identify the ability of pollen grains to induce gynogenesis and androgenesis. Indeed, as EMS mutagenized lines were used only as male in the crosses, only mutations carried by the male genome were tested. As female inducers are known like *ig* in maize (Huang and Sheridan, 1996; Evans, 2007) or the *haploid initiator* in barley (Mogensen, 1982), it could be interesting to explore the female genetic control of androgenesis and gynogenesis in *Arabidopsis* by running screens with the appropriate design.

This study suggests that the rate of mutations that can induce androgenesis/gynogenesis in *Arabidopsis* is very low. This should stimulate alternative approaches to obtain better knowledge of the genetic control of androgenesis and gynogenesis. It could be relevant to run such screens in another species, such as maize in which spontaneous mutants have been found. Further, this underlines the need of the identification and characterization of the few known loci that control these processes, such as *ggi1* in maize.
